# Risk factors and prevalence of human African trypanosomiasis in individuals living in remote areas of the republic of Congo

**DOI:** 10.1186/s12889-022-14577-9

**Published:** 2022-12-12

**Authors:** Viny Andzi Elenga, Abel Lissom, Darrel Ornelle Assiana Elion, Jeannhey Christevy Vouvoungui, Jean Claude Djontu, Reauchelvy Kamal Boumpoutou, Gabriel Ahombo, Francine Ntoumi

**Affiliations:** 1 Fondation Congolaise pour la Recherche Médicale, Brazzaville, Republic of Congo; 2 Faculty of Science and Technique, University of Marien Ngouabi, Brazzaville, Republic of Congo; 3 Department of Biological science, Faculty of Science, University of Bamenda, Bamenda, Cameroon; 4 Institute of Tropical Medicine, University of Tübingen, Tübingen, Germany

**Keywords:** Human African trypanosomiasis, Risk factors, Republic of Congo

## Abstract

**Background:**

Human African trypanosomiasis (HAT) is one of the world’s classical neglected tropical diseases representing a major public health threat in sub-Saharan Africa. Although the parasitic disease is in decline in the Republic of Congo, the better understanding of the epidemiological situation of active foci is required to reduce the risk of disease resurgence which could impede progress registered so far. The aim of this study was to determine the prevalence of HAT and the associated risk factors in individuals living in remote areas of the Republic of Congo.

**Methods:**

A cross-sectional survey was carried out in volunteers living in rural settings from June 2020 to January 2021. Socio-demographic and Clinical parameters of the participants were recorded. The presence of HAT-specific antibodies was assessed in whole blood, and then confirmed in serial diluted plasma samples using Card-Agglutination Trypanosomiasis Test (CATT)/*T.b. gambiense* CATT. The Capillary Tube Centrifugation (CTC) and Lymph nodes (LN) examination were done for detecting trypanosome parasites in CATT-serum positive cases. The staging of positive participants was determined by cerebrospinal fluid (CSF) examination.

**Results:**

Out of 8556 enrolled participants, 48.5% were more than 15 years old, 57.7% were unschooled and 67.2% practiced peasant activities. The prevalence of HAT infection was 0.3% with the predominance of patients at stage 1 of the disease (84.0%). The districts of Mindouli (OR: 25.9 (5.2–468); *p* = 0.0016) and Mpouya (OR: 13.3 (2.5–246); *p* = 0.0140) was revealed as the foci of high risk of HAT infection. Several factors were associated with an increased risk of HAT infection mainly including the non-schooling (OR: 5.1 (1.2–21.9); *p* = 0.0268), the life in couple or married (OR: 3.3 (1.0–11.3); *p* = 0.0545) and the practice of peasant activities (OR: 6.9 (2.4–29.3); *p* = 0.0017).

**Conclusion:**

This study highlights the need of revising and strengthening the strategies of HAT control in Republic of Congo, using an approach which will take into account the education level, the marital status and the occupation of the population at risk.

## Background

Human African trypanosomiasis (HAT) also called sleeping sickness, is a parasitic disease classified as one of the world’s classical “neglected tropical diseases” representing a major public health threat in sub-Saharan Africa [1]. Among the parasite species affecting the sub-Saharan Africa countries, *Trypanosoma brucei gambiense* (*T.b. gambiense*) is the most infectious in West and Central Africa [2] causing chronical form of the disease. *Trypanosoma brucei rhodesiense* (*T.b. rhodesiense*) is endemic in Eastern Africa and the pathogenic agent for the more acute form of the disease [3, 4]. In the last decade, the number of new cases fell below 10,000 for the first time in 50 years [1, 2]. However, the disease remains prevalent with a heterogeneous repartition across the continent. Over 60% of HAT cases have been reported in the Democratic Republic of Congo which bears the highest burden of the disease [1, 5]. Important progress has been achieved in the management of *gambiense* HAT cases in endemic areas including the development of new rapid diagnostic tests used for mass and passive screening, the introduction of new treatments for both stages 1 and 2 of the disease [6, 7]. Despite to the presence of few active foci in several countries, the potential for outbreaks and disease resurgence is still at a high risk. This is due to the circulation of *T. b. gambiense* in both human and animal species [8, 9], movement of individuals in and out of endemic settings, and the presence of competent Tsetse fly in most epidemiological settings [10].

In Republic of Congo, the disease is in decline with a national prevalence of 0.1% [11]. According to the latest report of the National Control Program for human African trypanosomiasis of the Republic of Congo, only the foci of savannah and of corridor (Congo River) are still active with regular transmission of HAT. The localities of Loudima, Ngabe, and Mpouya are at the forefront of the transmission [11]. A significant decrease of HAT transmission has been reported across the country, may be due to the results of improved case detection, management and vector control activities and the improvement of infrastructure like roads [12]. However, there is a still great challenge in determining the real epidemiological situation of HAT infections in remote areas, mainly the villages of the foci at risk due to the difficulty of access. A systematic survey of HAT cases as well as factors related to the perennation of the disease in these hotspots is thus required. Understanding of the epidemiological situation of the HAT in endemic foci will contribute to the strengthening of the strategy eradication, and to avoid resurgence of the disease impeding the progress recorded so far. The aim of this study was to determine the prevalence of HAT and the associated risk factors in individuals living in remote endemic areas of the Republic of Congo.

## Methods

### Study areas

The study was carried out in rural areas in four districts of the republic of Congo, including Mpouya (in plateaux Department), Mindouli (Department of Pool), Mossaka and Loukolela both located in the Department of Cuvette. These districts are predominated by a tropical savanna climate. Mpouya is a region located at 2°36′57″South, 16°12′43″East with an altitude of 350 m. It is located near the Congo River and comprises 9000 inhabitants mainly involved in agriculture and fishing activities. Mindouli is geo-localized at 4° 16′ 24″ South, 14° 23′ 2″ East. It accounts for 53,584 inhabitants and agriculture is the main economic activity of the region. The district of Mossaka, located at 1° 13′ 53″ South, 16° 46′ 58″ East, accounts for 25,636 inhabitants. It is bounded to the east by the Democratic Republic of Congo and fishing is the main economic activity the locality. The Loukoléla district is geo-localized at 1° 3′ 58″ South, 17° 10′ 6″ East, and has 21,442 inhabitants. It is located on the banks of the Congo River, at about 50 km upstream from the confluence between the Congo and the Sangha. One of the main economic activities in this district is fishing and smoking of fish which is transported to the main consumption centres of the country.

### Study design

A cross-sectional study was conducted during a mass diagnostic campaign from June 2020 to January 2021. The population of interest included the participants of all age who lived in the study areas for at least 3 months. During the mass diagnostic in each district, we conducted a simple randomized sampling.

According to the sample size calculator Raosoft 2004, a sample size of 3709 participants was estimated (with a target of having at least 1/4 participants per district, which correspond to 928 participants/district), assuming that the total size of population of the study areas was 109,662, a confidence level was fixed at 95%, an error margin of 0.1% and a prevalence of HAT in Republic of Congo of 0.1% (reference). The below Formula was used to determine the sample size:

$$\textrm{n}=\textrm{N}\times \frac{\frac{{\textrm{Z}}^2\times \textrm{p}\times \left(1-\textrm{p}\right)}{{\textrm{e}}^2}}{\textrm{N}-1+\frac{{\textrm{Z}}^2\times \textrm{p}\times \left(1-\textrm{p}\right)}{{\textrm{e}}^2}}$$

Where **n** is the sample size; **e** is the marginal error, **N** is the population size of the locality; **p** is the prevalence of HAT. and **z** is the confidence level.

After obtaining the ethical clearance and the administrative authorizations, the participants were randomly enrolled in the study sites for blood sample collection. A consent form was provided by the participants or the parent of minor (< 18 years) participants. The sociodemographic and clinical parameters of the participants were recorded in a well-structured collection sheet (age, gender, school level, occupation etc). EDTA-blood (3 ml) and cerebrospinal fluid (CSF) were collected by a nurse, and transported to the Reference health Centre of each district for further processing. HAT screening was done according to the Algorithm of the national Program of HAT control in Republic of Congo. The first test was done using the card agglutination test for trypanosomiasis (CATT) in whole blood and plasma samples of each participant. The Capillary Tube Centrifugation (CTC) technique was used as additional test for detecting trypanosoma parasites in participants with a positive CATT. The HAT positive cases were treated either with stage 1 or stage 2 protocol based on the results of the cerebrospinal fluid (CSF) examination, according to National Control Program for HAT of Republic of Congo.

### Diagnosis of human African Trypanosomiasis

The diagnostic of HAT was based on active screening of the infection. Results of antibody and parasite detection were necessary to make the final decision of participant clinical status.

### Titre antibody screening

CATT/*T.b. gambiense* was used for the detection of the *T.b. gambiense* specific antibody in sample as described by the manufacturers [13]. Briefly, the undiluted blood samples of each participant were firstly tested for HAT antibodies. The participants with a positive result were confirmed following antibody detection titration-method in plasma samples using the CATT. The titre (highest dilution giving agglutination) was determined. Participants with titres < 1∶8 were considered CATT negative, while titres ≥1∶8 were considered CATT positive.

### Parasite detection and classification of HAT stages

The Capillary Tube Centrifugation (CTC) and Lymph nodes (LN) examination were used as additional test for detecting trypanosome parasites in participants with a positive CATT. All participants positive with CATT but negative with CTC were considered as healthy carriers [8, 14]. The presence of trypanosome and the number of white blood cells in the CSF were assessed to classify the disease progress of positive participants. The screening of the parasite was done after centrifugation of CSF immediately after lumbar puncture [15]. The disease progress was classified as first stage when no trypanosomes were detected in the CSF and the WBC count was ≤5 WBC/μl. The second stage of the disease was defined by the presence of trypanosomes in the CSF with > 5 WBC/μl.

### Data analysis

Data management and tabulation were carried out using EpiInfo7 version 7.2.2.6 and Microsoft Excel 2010. All statistical analyses were done using R version 3.6.3 (2020-02-29) and RStudio Version 1.2.5033 software. The normality of data distribution was checked using the Shapiro–Wilk test [16]. Qualitative data were expressed as percentages, and quantitative data by medians (with range) or mean (standard deviation). Pearson’s Chi-square test (or Fisher’s exact tests when appropriate) was used to compare percentages between groups. The strength of association between each of the potential risk factors and the occurrence of HAT was calculated using univariate logistic regression using the complete cases. The odds ratios (OR) are given with a 95% confidence interval (CI). The statistical significance threshold for the tests was set at 5%.

### Ethical consideration

This study received an ethical approval from the Institutional Ethics Committee of *Fondation Congolaise pour la Recherche Medical* (Ethical Clearance N° 023/CIE/FCRM/2019) and the administrative authorisations from each mayor of the areas. All experiments were performed in accordance with relevant guidelines and regulations. Prior to enrolment, participants were informed in writing and orally about the study and its benefits. A written informed consent form was obtained from the adult participants for participating in the study. For the minor (< 18 years) a written informed consent form was obtained from their parent/guardian. In addition, a written assent form was obtained from children aged between 15 to 17 years hold.

## Results

### General characteristic of the study population

A total of 8556 participants were enrolled in this study (Table 1), coming from four districts including Loukolela (39.1%), Mindouli (22.9%), Mossaka (11.5%) and Mpouya (26.5%). The participant’s age ranged between 0 and 94 years, with a median age of 14 years (IQR: 10–34 years). Male participants were more represented (52.8%) in the study population compared to female. Among the participants with eligible age for schooling or marriage, the school level was highly represented by the group of unschooled participants (57.7%), while participants living in couple (61.4%) was the predominant group regarding the marital status. Most of the participants were either farmer (32.8%), student (32.7%) or fisher (man) woman (34.4%).

**Table 1:** Characteristic of the study population

Sociodemographic characteristic	Number (***N*** = 8556)	Percentages (%)
**District**		
Loukolela	3351	39.1
Mindouli	1956	22.9
Mossaka	981	11.5
Mpouya	2268	26.5
**Age (years), Median (Range)**	14 (0–94)	
**Age groups (years)**	**(N = 8556)**	
[0–11]	2892	33.8
[12–14]	1509	17.6
[≥15]	4145	48.5
Missing	10	0.1
**Gender**	**(N = 8556)**	
Female	4029	47.1
Male	4514	52.8
Missing	13	0.1
**School level**	**(*N*** **= 7774)**	
Unschooled	4488	57.7
Primary	2087	26.9
Secondary	1199	15.4
**Marital status**	**(*N*** **= 5547)**	
Single	1881	33.9
Divorcee	19	0.3
As a couple or married	3405	61.4
Widower	21	0.4
Missing	221	4.0
**Profession**	**(N = 8556)**	
Farmer	2111	32.8
Student	2108	32.7
Teacher	4	0.1
Fisher (man) woman	2219	34.4

*N* Total number of enrolled participants, *CI* Confidence Interval

### Human African trypanosomiasis diagnostic

**Fig. 1:**
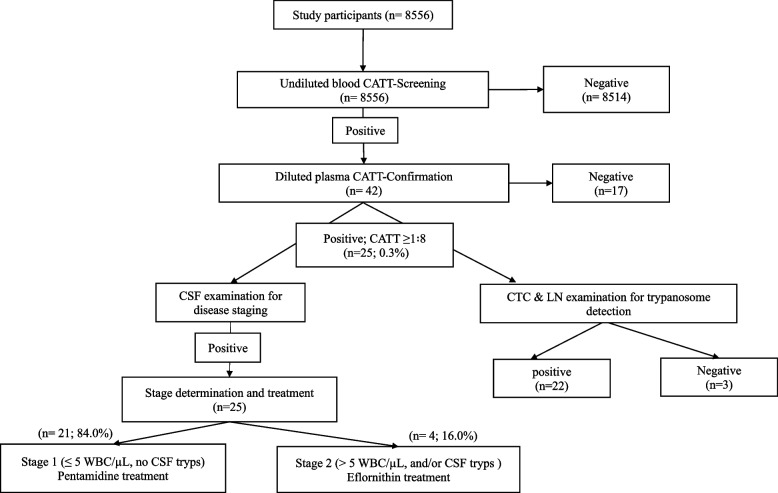
Outcomes of Human African Trypanosoma Infection in the study population. CATT: card agglutination test for trypanosomiasis. CSF: cerebrospinal fluid. Tryps: Trypanosoma. CTC: The Capillary Tube Centrifugation. LN: Lymph node. WBC: white blood cell. CSF tryps: presence of Trypanosoma in cerebrospinal fluid

The results of the HAT diagnostic showed that the prevalence of this disease was 0.3% (25/8556) in the study population (Fig. 1). Among the positive participants, 84.0% (21/25) were at stage 1 of the disease.

### Sociodemographic predictors of human African trypanosomiasis infection

The relationship of the participant characteristics with the presence of status of HAT infection was investigated in this study and the results were reported in Table 2. The distribution of the HAT infected participants among the study site showed that, the districts of Mindouli (OR: 25.9 (5.2–468); *p* = 0.0016) and Mpouya (OR: 13.3 (2.5–246); *p* = 0.0140) were the foci at high risk of infection with important prevalence. Among the age groups, participants aged more than 15 years were significantly at high risk (OR: 7.4 (2.2–46.0); *p* = 0.0070) of HAT infection compared to the other age groups. HAT infection was significantly associated with the school level of the participants (*p* = 0.003), with a higher risk of infection observed in the group of the unschooled participants (OR: 5.1 (1.2–21.9); *p* = 0.0268), compared to other the groups with eligible age for schooling. Regarding the marital status of the participants with eligible age for marriage, there was a significant association(*p* = 0.0444) between this parameter and the HAT infection, with a higher risk of infection observed in participants living in couple or being married (OR: 3.3 (1.0–11.3); *p* = 0.0542).

**Table 2:** Association of HAT infection with sociodemographic characteristics

Socio-demographic characteristic	HAT status		Statistical test (***p***-value)	Univariate logistic regression		
	Negative, n (%)	Positive, n (%)		OR	95% CI	p-value
**Districts**						
Loukolela	3350 (99.97)	1 (0.03)	0.0001^*1*^	1.0	–	
Mindouli	1941 (99.2)	15 (0.8)		25.9	5.2–468	**0.0016**
Mossaka	981 (100)	0 (0)		0.0		0.9885
Mpouya	2259 (99.6)	9 (0.4)		13.3	2.5–246	**0.0140**
**Age groups (years)**						
[0–11]	2890 (99.9)	2 (0.1)	0.0012^*2*^	1.0	–	
[12–14]	1507 (99.9)	2 (0.1)		1.9	0.2–16.0	0.5152
[≥15]	4124 (99.5)	21 (0.5)		7.4	2.2–46.0	**0.0070**
**Gender**						
male	4504 (99.8)	10 (0.2)	0.1978^*1*^	1.0	–	
female	4014 (99.6)	15 (0.4)		1.7	0.8–3.9	0.2028
**School level**						
Primary	2085 (99.9)	2 (0.1)	0.003^*1*^	1.0	–	
Unschooled	4466 (99.5)	22 (0.5)		5.1	1.2–21.9	**0.0268**
Secondary	1199 (100)	0 (0)		0.3	0.0–7.2	0.4954
**Marital status**						
single	1878 (99.8)	3 (0.2)	0.0444^*2*^	1.0	–	
Divorcee	19 (100)	0 (0)		13.8	0.7–275.5	0.0864
As a couple or married	3387 (99.5)	18 (0.5)		3.3	1.0–11.3	**0.0542**
Widower	21 (100)	0 (0)		12.5	0.6–249	0.0984
**Profession**						
Fisher (man) woman	2211 (99.6)	8 (0.4)	0.0004^*2*^	1.0	–	
Farmer	2097 (99.3)	14 (0.7)		1.9	0.8–4.6	0.1679
Student	2108 (100)	0 (0)		0.0		0.9871
Teacher	4 (100)	0 (0)		0.0		0.9994

*n (%)* number of individuals (percentage of individuals), *OR* Odds Ratio, *CI* Confidence Interval

^1^Pearson’s Chi-squared test

^2^Fisher’s exact test

### Association of HAT infection with peasant activities, knowledge and communication action used on HAT

The results of the relationship of HAT infection with peasant activities of the participant of this study was reported in Table 3. It was shown individual practising peasant activities were at a high risk of HAT infection (OR: 6.9 (2.4–29.3); *p* = 0.0017). However, no relationship was found between HAT infection and the frequency on the practice of peasant activities (*p* > 0.9999), communicational action (*p* = 0.4351) or knowledge of the disease (*p* = 0.4274).

**Table 3:** Association of HAT infection with peasant activities, knowledge and information about HAT

Sociodemographic characteristic	HAT status		Statistical test (p-value)	Univariate logistic regression		
	Negative n (%)	Positive n (%)		OR^*2*^	95% CI^*2*^	p-value
**Practice of peasant activities**						
No	4140 (99.9)	3 (0.1)	0.0003^*1*^	1.0	–	
Yes	4384 (99.5)	22 (0.5)		6.9	2.4–29.3	**0.0017**
**Regular practice of peasant activities**						
Often (≤1/ week)	369 (99.7)	1 (0.3)	0.9999^*2*^	1.0	–	
Always (> 2/week)	4045 (99.5)	21 (0.5)		1.9	0.4–34.4	0.5259
**Knowledge about HAT**						
No	5593 (99.7)	14 (0.3)	0.4274^*1*^	1.0	–	
Yes	2938 (99.6)	11 (0.4)		1.5	0.7–3.3	0.3184
**Communication action used on HAT**						
Media	950 (99.9)	1 (0.1)	0.1274^*1*^	1.0	–	
school	1 (100)	0 (0)		0.0		0.9968
Missionary	2 (100)	0 (0)		0.0		0.9954
Sensitisation	1985 (99.5)	10 (0.5)		4.8	0.9–8.8e+ 01	0.1358

*n (%)* number of individuals (percentage of individuals), *OR* Odds Ratio, *CI* Confidence Interval

^1^Pearson’s Chi-squared test

^2^Fisher’s exact test

### Relationship of HAT infection with clinical signs of the participant

The association of HAT infection with the health state of the participants was determined in this study and reported in Table 4. Three clinical signs were recorded from the study population, including Inoculation canker, sleep disorders and trypanids. Most of the participants had no sign related to HAT. There was a significant association between the presence of clinical signs and the HAT infection (*p* < 0.0001), with risk of the disease being strongly associated to sleep disorders (OR: 426.4 (75.1–2.4e+ 03); < 0.0001). However, no relationship was observed between the HAT infection and the mental state of the participant in this study (*p* > 0.9999).

**Table 4:** Association of HAT infection with observed clinical sign and mental health state

Sociodemographic characteristic	HAT status		Statistical test	Univariate logistic regression		
	Negative n (%)	Positive n (%)		OR	95% CI	p-value
**Clinical sign observed from the HAT**						
No sign	8527 (99.8)	20 (0.2)	< 0.0001^*2*^	1.0	–	
Inoculation canker	1 (100)	0 (0)		0.0		0.9948
Sleep disorders	3 (50.0)	3 (50.0)		426.3	75.1–2.4e+ 03	**< 0.0001**
Trypanids	0 (0)	2 (100)		2.5e+ 09	0.00 - NA	0.9832
**Mental health status**						
Normal	8519 (99.7)	25 (0.3)	> 0.9999^*2*^	1.0	–	
Obsessed	2 (100)	0 (0)		0.0		0.9888

HAT status refers to the biological status

*n (%)* number of individuals (percentage of individuals), *OR* Odds Ratio, *CI* Confidence Interval

^1^Pearson’s Chi-squared test

^2^Fisher’s exact test

## Discussion

The Republic of Congo is among countries still affected by HAT, with HAT-*gambiense* being the predominant species. Regular transmission of the disease occurs in five of the twelve departments (Bouenza, Niari, Pool, Plateau, and Cuvette) which are located in the savannah and the corridor (Congo river) of the country [11]. The present work aimed to determine the prevalence and risk factors in remote areas of the Republic of Congo, including the villages of the districts of Mpouya (in plateaux Department), Mindouli (Department of Pool), Mossaka and Loukolela (both located in the Department of Cuvette).

The prevalence of the human African trypanosomiasis was 0.3% in the study population, with the majority of patients being in stage 1 of the disease. This prevalence is slightly higher than what has been reported by the national control programme of HAT of Congo (0.1%) in 2019 [11]. A retrospective study conducted in children undergoing routine HAT diagnostic in Brazzaville between 2004 and 2020 reported a prevalence of 2.7% corresponding to an annual prevalence of 0.17%. The majority of the positive cases in this study came from the districts Ngabe, Mossaka and Mpouya [17]. So, the link between the late onset of clinical signs (neurological stage) and the presence of infection which could undermine a passive approach to HAT control. The low annual prevalence of HAT reported in the previous study compared to our study may be due to the study design used. First; the recruitment of the participants in the previous study was affected by frequency of appointment of the patient in the health centre during 16 years (2004 to 2020). Secondly, this study was focused in children, reducing the probability of having positive cases. In contrary, we conducted a mass diagnostic targeting the participants of all age groups. Furthermore, we found that the participants aged more than 15 years were more infected by HAT compared to the other age groups. HAT cases was significantly predominant in Mindouli and Mpouya districts, with a high risk of the infection, confirming what has been reported previously [12].

Many studies reported that the *Glossina* species identified as the main vector of the *T. b. gambiense* infection in Congo feed on both human and animal and always display infectivity [12]. All this suggests that more efforts are still needed for the eradication of the disease in these districts, both in human and animal population.

The relationship between the risk of the HAT infection and vector control has been well documented, but little is still known concerning the human behaviour and perennation of this disease. In this work, we determined the factors associated with the risk of HAT infection in the overall study population. It was shown that, unschooled participants as well as those aged more than 15 years were significantly at high risk of HAT infection compared to their counterpart groups. This suggest that education is a key tool in the fighting strategy against outbreak of this disease. In fact, the unschooling is one of the indicators of underdevelopment that might be associated with a decrease of professional performance, and therefore with the socioeconomic development in a given region [18]. In addition, older participants are more likely to be involved in peasant activities (agriculture and fishing) and thus at higher risk of the HAT infection. Our findings showed a significant association between practice of peasant activities and the high risk of HAT infection; regardless of the regularity. It was reported that the rate of HAT infected children is generally less than half of that observed in adults because of their lower exposure to flies during daily activities [19]. In addition, the majority of infected children are those who often accompany their parents during hunting or fishing activities as notified in the DRC, the most endemic country [1]. No relationship was observed between sex and the risk of HAT infection. This might be justified by the fact that, in areas of transitional vegetation in forest and wooded savannah, where infection is linked to agricultural work or activities around water points, the prevalence is similar in both sexes [19, 20].

A significant association was found between the marital status of participants and the HAT infection in this study, with a higher risk of infection observed in participants living in couple or being married. This suggests that the proximity of the human beings might plays an important role during the transmission of the disease by the tsetse-fly. Beside human-human transmission via the vector, a possible Human-Animal transmission was suggested, since the presence of *T. b. gambiense* in animals has been demonstrated in several studies [21, 22].

The association of HAT infection with health state of the participants was also investigated in this study. Three main clinical signs were recorded from the study population, including Inoculation canker, sleep disorders and trypanids. Although the majority of the infected participants did not present any sign related to HAT, sleep disorders was the major HAT-related sign developed by patients of this study. This result is in line with what has been reported by Ibara et al. during a retrospective study conducted in Bazzaville [17]. Somnographic studies conducted by Buguet and Damba showed that HAT disrupts the circadian rhythm of the sleep-wake cycle resulting in the sleep fragmentation rather than the often described inversion [23]. However, no relationship was observed between the HAT infection and the mental state of the participant in this study.

The fact that this work was conducted on a large population constitutes one of the strengths of the study. Moreover, the participants came from remote endemic areas that are difficult to access. However, one limitation of the study is the lack of information about domestic animals during the survey which could be an important factor for HAT transmission in these areas.

## Conclusion

This study highlights the need of strengthening the strategies of fighting against the HAT. This could be done by using an approach which will take into account the education level, the marital status and the occupation of the population at risk. In addition, a continuous mass diagnostic and treatment of the infected individuals, as well as the vector control may strengthen HAT intervention measures, and then prevent its resurgence in high-transmission settings.

## Data Availability

All data are fully available without restriction. Data are available from the FCRM Institutional Data Access. All request for Data should be addressed to the Executive Director of FCRM reachable by the following address Prof. Francine Ntoumi. Villa D6-Cité OMS-Djoué – Brazzaville- République du Congo. Tel. + 242 06997 79 80. Email: francine.ntoumi@uni-tuebingen.de
